# Mnemonic-trained brain tuning to a regular odd-even pattern subserves digit memory in children

**DOI:** 10.1038/s41539-023-00177-8

**Published:** 2023-08-11

**Authors:** Yafeng Pan, Ning Hao, Ning Liu, Yijie Zhao, Xiaojun Cheng, Yixuan Ku, Yi Hu

**Affiliations:** 1 Shanghai Key Laboratory of Mental Health and Psychological Crisis Intervention, School of Psychology and Cognitive Science, East China Normal University, Shanghai, China; 2 Department of Psychology and Behavioral Sciences, Zhejiang University, Hangzhou, China; 3 The State Key Lab of Brain-Machine Intelligence, Zhejiang University, Hangzhou, China; 4 School of Psychology, Hainan Normal University, Haikou, China; 5 Institute of Science and Technology for Brain-Inspired Intelligence, Fudan University, Shanghai, China; 6 School of Psychology, Shenzhen University, Shenzhen, China; 7 Guangdong Provincial Key Laboratory of Brain Function and Disease, Department of Psychology, Sun Yat-sen Unviersity, Guangzhou, China; 8 Peng Cheng Laboratory, Shenzhen, China

**Keywords:** Working memory, Human behaviour

## Abstract

It is said that our species use mnemonics – that “magic of memorization” – to engrave an enormous amount of information in the brain. Yet, it is unclear how mnemonics affect memory and what the neural underpinnings are. In this electroencephalography study, we examined the hypotheses whether mnemonic training improved processing-efficiency and/or altered encoding-pattern to support memory enhancement. By 22-day training of a digit-image mnemonic (a custom memory technique used by world-class mnemonists), a group of children showed increased short-term memory after training, but with limited gain generalization. This training resulted in regular odd-even neural patterns (i.e., enhanced P200 and theta power during the encoding of digits at even- versus odd- positions in a sequence). Critically, the P200 and theta power effects predicted the training-induced memory improvement. These findings provide evidence of how mnemonics alter encoding pattern, as reflected in functional brain organization, to support memory enhancement.

## Introduction

The use of mnemonic techniques or strategies that aid information storage is one of the extraordinary skills demonstrated by humans. Spontaneous use of mnemonics is ubiquitous; examples include basic rehearsal (e.g., “apple/apple/…”) and homophonic association (e.g., see/sea). Besides, human memory also benefits from the training of the Method of Loci^1^ and the digit-image method^2^, the quintessential mnemonics employed by world-class mnemonists. Such training-induced boost occurs at various levels ranging from memory performance^3^ to functional brain organization^4^, and involves multiple populations from developing children^5^ to the elderly^6^.

There are two hypotheses accounting for the effectiveness of mnemonic training. One is the so-called *processing-efficiency* hypothesis, which holds the view that the cognitive processing of to-be-memorized materials consumes less cognitive resource after training. This is based on the fact that mnemonists go through extensive training and accumulate abundant memory experiences; as a result, individuals’ encoding becomes less effortful and their neural activity generally decreases^7^. Specifically, the lower levels of neural dynamics (e.g., ERP amplitude, oscillatory power) were associated with more efficient and better performance^8^; that is, cognitive performance showed negative associations with the neural activity. The experience-induced efficiency at the neural level has been found in domain-specific expertise, such as recognition of cars^9^ and recall of complex narratives^10^.

As an alternative, the *encoding-pattern* hypothesis proposes the alteration of the encoding pattern after mnemonic training. This is inferred from the previous studies on world-class mnemonists who were proficient in the digit-image method^2,11–13^. For example, they segmented the numeric sequence “241472” into digit combinations of “24”, “14”, and “72”, and further visualized into images as “*lion*”, “*key*”, and “*penguin*”. The digits were presented sequentially and the participants controlled the rate of presentation of the digits. The digits were shown until the participants coded them as images. There was no time limit for the encoding of a given digit. Results revealed that, more encoding time was spent on even-position digits than odd-position digits^11,12^. This was due to that participants were only able to map the 2-digit number to an image at the position of even-digits, according to our early investigations^11,12^. Because the digits were presented sequentially, participants had more time to process and create a mental image for the even-digit than the odd-digit, resulting in a stronger association between the even-digit and the final image. Previous studies have extensively investigated brain activity during tasks that involve differences in encoding or reaction times^14–16^; therefore, we speculated that there would be distinct neural responses to odd- and even-position digits. This difference may be attributed to the strategy employed by mnemonists, which involves generating or associating a mental image immediately upon the presentation of each even-position digit. This conjecture has been supported by preliminary findings of differential electroencephalographic (EEG) responses, such as a higher right central P200 for encoding digits on even- versus odd- positions^13^.

In the context of mnemonic training, the processing-efficiency and encoding-pattern hypotheses can be mapped onto (1) training aimed at improving memorization of a certain perceptual category and (2) training focused on enhancing associative memory, respectively. Within the broader context of learning, mnemonic training can lead to expertise improvement (e.g., digit memory) in a general sense, or it can shape the process of memorization in a specific learning task^8,9,13^. If the training generally enhances memorization (domain-general), it may result in less intense neural activity required for encoding digits^7,8^. In contrast, if it specifically alters the digit encoding pattern (domain-specific), this behavioral or cognitive change is expected to be reflected at the neural level as well^13–15^.

**Fig. 1:**
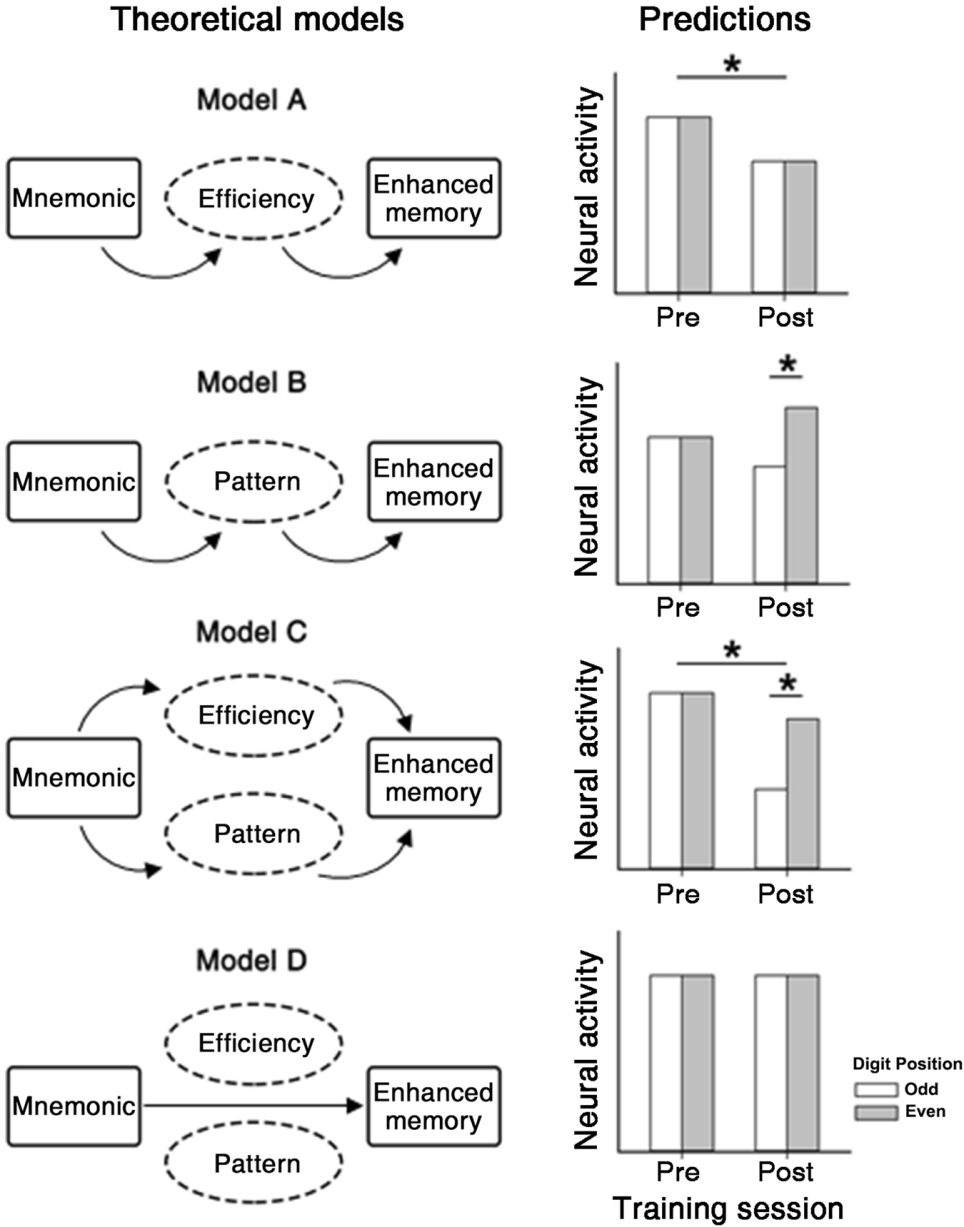
Theoretical models. Predictions for neural activity at post-training compared with pre-training. Model A expects generally decreased neural activity at post-training (vs. pretraining); Model B predicts differential neural activity between digit position odd and even; Model C, integrating Model A and Model B, anticipates differential neural activity during processing digits at position odd versus even and generally decreased neural activity after training; Model D predicts no difference in terms of training sessions and digit positions. The asterisk denotes a hypothetical difference between conditions.

In light of the two hypotheses above, we propose four theoretical models to depict neural activity concerning encoding patterns (position odd vs. even) from pre-training to post-training (Fig. 1). Four models further derive EEG predictions as follows. Model A, based on the processing-efficiency hypothesis, proposes that mnemonic training generally decreased neural activity at post-training compared with pre-training. Model B, based on the encoding-pattern hypothesis, expects the differential neural activity during processing digits at position odd versus even; this occurs at post-training but not pre-training. Model C, integrating the previous two models, anticipates the differential neural activity both for training sessions and digit positions; specifically, it expects differential neural encoding activity at digit position odd versus even in the post-training session, and a lower level of neural activity at post-training than that at pre-training. Finally, model D expects no difference for digit positions and training sessions.

To test those models, we recruited a group of developing children who were trained to use the digit-image mnemonic within 22 days. Developing children with low memory capacity typically make poor educational progress, and it has been speculated that difficulties in efficiently encoding information may be a contributory factor^17^. Intense mnemonic training has been demonstrated to boost performance in children^5,18,19^. For instance, Turley-Ames and Whitfield^19^ demonstrated that the rehearsal strategy training improved working memory, and memory gains extended to higher cognitive functions such as reading ability. At the neural level, despite previous studies have investigated the neural changes following mnemonic training in children using neuroimaging techniques^20–22^, it remains less clear that how mnemonic training reshapes neural dynamics during encoding in children.

There is currently no consensus on the temporal dynamics that underlie the encoding processes of individuals who use the digit-image mnemonic. However, because the digit-image mnemonic shares several characteristics with the visual imagery mnemonic discussed in prior studies^23^, it is believed that EEG brain signals such as P200 could be implicated. The P200 component, which is associated with attention during mental imagery tasks, was found to be enhanced during an auditory imagery task^24^, possibly reflecting the early top-down allocation of attention. Additionally, superior mnemonists who used the digit-image mnemonic to encode digit sequences showed P200 activity^13^. The theta rhythm has also been implicated in various functions related to human memory^25,26^ and mental imagery^27,28^, both of which are essential processes for using the digit-image mnemonic. For example, a study using simultaneous EEG-fMRI observed that theta power during encoding predicted subsequent memory performance and default mode network deactivation^26^. Frontal theta activity was also observed to increase during motor and visual imagery of scenes, which may reflect increased cognitive effort^27,28^.

In the current study, we tested the hypotheses (and the derived models) that mnemonic training improves processing-efficiency and/or alters encoding-pattern to support memory enhancement in developing children. A short-term digit memory task was used to record EEG data and to estimate the performance increment. We predicted that a 22-day mnemonic training was sufficient to trigger striking enhancement in memory. Moreover, we expected that our training would induce changes in P200, which was involved in the use of mnemonic and digit processing^13^. In addition, we anticipated that mnemonic training would alter theta power; this anticipation was based on previous research showing theta power during encoding was a robust index predicting subsequent memory performance^26^. Finally, a battery of cognitive tests was applied to explore whether the mnemonic training led to generalized enhancements in other cognitive abilities.

## Results

**Fig. 2:**
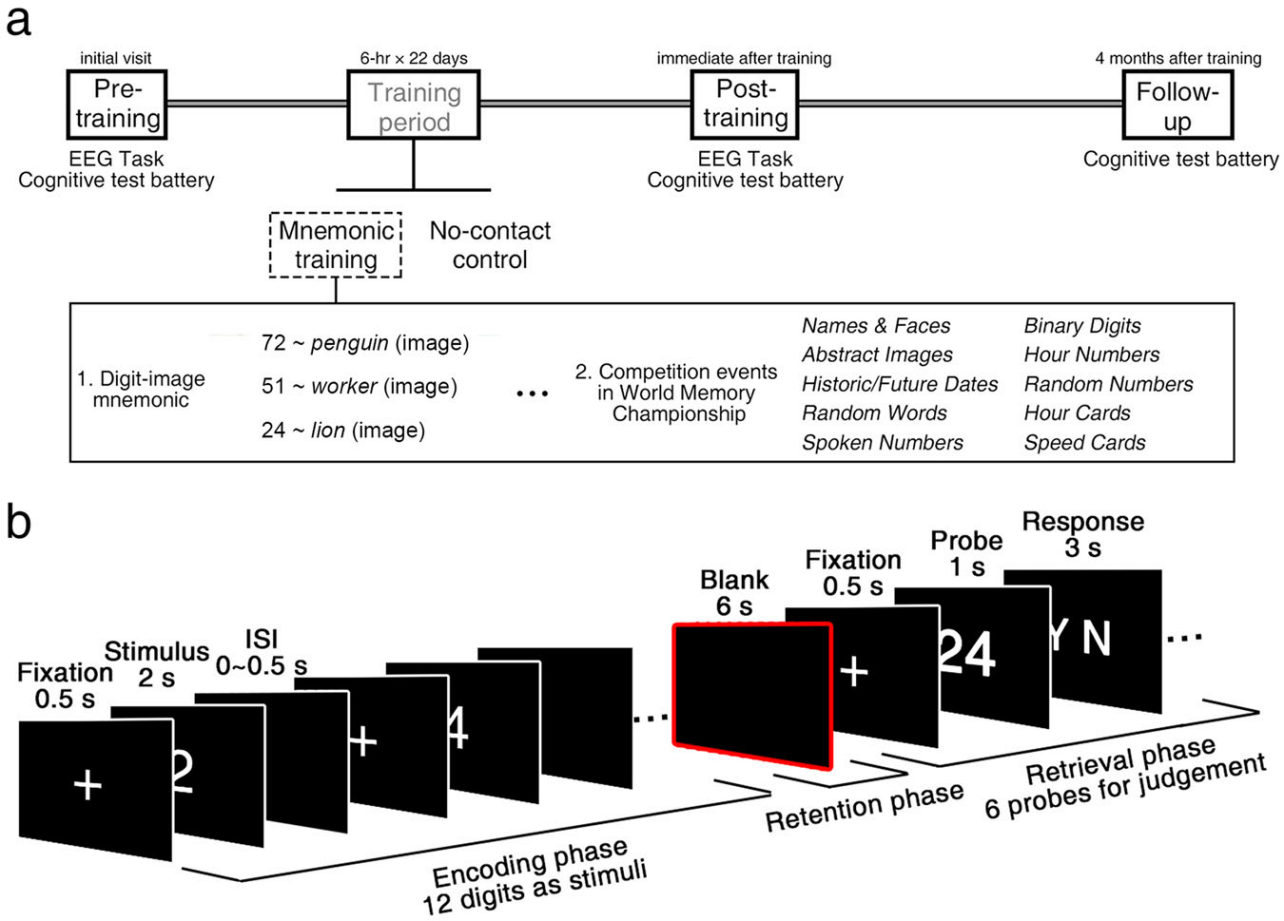
Visualization of experimental sessions and task. **a** There were four sessions, entailing pre-training, training, post-training, and follow-up tests. During the training session, children were trained with the digit-image mnemonic, which was further practiced in competition events (see Methods for details). **b** The time flow of the EEG task. There were 20 blocks in total. Each block entails encoding, retention, and retrieval phases.

To investigate how mnemonic training affects neural dynamics during encoding in children, we recruited a group of developing children. Participants were divided into two groups: a mnemonic training (MT) group and a no-contact control (NC) group based on their interests. Both groups underwent four sessions (Fig. 2a): (1) pre-training tests (initial visit), where participants’ brain responses to digit sequences and their cognitive abilities were measured using an EEG task (Fig. 2b) and a cognitive test battery, respectively (see Methods for detailed descriptions of the tasks); (2) a training period lasting 22 days, during which the MT group was taught how to use the digit-image method on different materials while the NC group received no training; (3) post-training tests (immediately after training) to evaluate the effects of the training using the same tasks administered during the initial visit; (4) follow-up tests (conducted 4 months after training) to estimate the training effects on some measures (i.e., digit matrix and number-noun pairs) in the cognitive test battery again.

### Behavioral results

#### EEG task

**Fig. 3:**
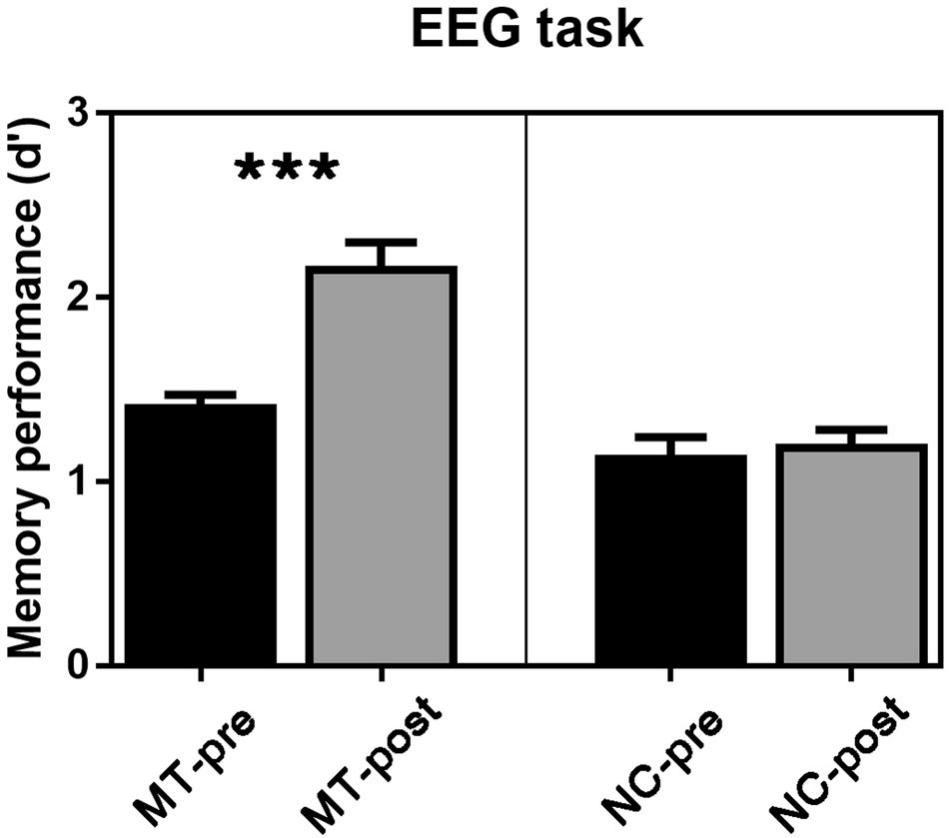
Behavioral results of the EEG task. In the MT group, memory performance (d') was higher for the post-training phase than for the pre-training phase. In the NC group, there was no session difference. NC-pre/NC-post = non-contact group at pre-/post-training, MT-pre/MT-post = mnemonic training group at pre-/post-training. ***p < 0.001, two-sided, paired-sample *t*-test. Error bars indicate standard error.

A two-way ANOVA with a between-subject factor of Group (MT vs. NC) and a within-subject factor of Session (pre- vs. post-training) was conducted on the performance of the EEG task. It revealed the significance only for the Group × Session interaction, *F* (1, 31) = 15.46, *p* < 0.001, *η*^*2*^_*partial*_ = 0.30. Upon further analysis, it was found that post-training discriminability (*M* ± *SE*, 2.10 ± 0.19) was significantly higher than pre-training discriminability in the MT group (1.37 ± 0.12), *p* < 0.001. However, there was no significant difference between the pre-training phase (1.12 ± 0.15) and the post-training phase (1.16 ± 0.10) in the NC group, *p* > 0.05 (Fig. 3).

#### Cognitive battery tests

Next, we assessed whether mnemonic training led to the generalized gain of cognitive abilities (this entailed both near-transfer, e.g., working memory, and far-transfer, e.g., divided attention). We conducted a series of ANOVAs on each measure of our cognitive test battery, with Group as a between-subject factor and Session as a within-subject factor. After applying false-discovery-rate (FDR) corrections to all resulting *p* values, we observed a significant interaction effect on the *number-noun pairs* measure, suggesting that the MT group performed better on this test compared to the NC group after undergoing training and the improvement persisted over time as seen in the follow-up (Table 1). No other significant interaction effects were observed. These findings indicate a limited enhancement in the assessed cognitive abilities for the MT group compared with the NC group after training. Supplementary Table 1 contains a comprehensive set of ANOVA results.

**Table 1:** The performance on cognitive test battery (*M* ± *SE*).

Test battery	Pre-training session		Post-training session		Follow-up session	
	MT	NC	MT	NC	MT	NC
Perceptual speed (ms)						
Choice reaction-digit	664.28 (34.81)	630.62 (28.36)	631.62 (22.92)	634.58 (31.00)		
Choice reaction-figure	967.65 (75.80)	1011.06 (81.82)	686.28 (62.42)	694.74 (46.18)		
Inhibitory control (ms)						
Stroop color	305.42 (71.02)	315.66 (53.89)	185.14 (29.43)	172.63 (35.09)		
Working memory (accuracy)						
Digital 2-back	0.86 (0.03)	0.80 (0.03)	0.90 (0.02)	0.85 (0.03)		
Spatial 2-back	0.85 (0.03)	0.81 (0.03)	0.89 (0.02)	0.85 (0.03)		
Short-term memory (accuracy)						
Digit matrix	0.71 (0.04)	0.55 (0.04)	0.90 (0.03)	0.64 (0.04)	0.80 (0.05)	0.66 (0.06)
Episodic memory (accuracy)						
Word lists	0.32 (0.09)	0.36 (0.05)	0.57 (0.14)	0.33 (0.03)		
Number-noun pairs	0.21 (0.03)	0.22 (0.01)	0.47 (0.06)	0.12 (0.02)	0.55 (0.08)	0.19 (0.04)
Reasoning ability (accuracy)						
Reasoning	0.78 (0.07)	0.80 (0.03)	0.80 (0.05)	0.85 (0.02)		
Spatial imagination (accuracy)						
Rotations	0.87 (0.08)	0.84 (0.08)	0.86 (0.10)	0.85 (0.09)		
Divided attention (accuracy)						
Multiobject tracking	0.80 (0.06)	0.79 (0.06)	0.92 (0.02)	0.90 (0.01)		

*MT* the mnemonic training group, *NC* the no-contact control group.

### Electrophysiological results

To examine how mnemonic training (MT) alters neural dynamics during encoding in children, we used a planned contrast-based ANOVA approach. Such a pre-planned approach was extrapolated from our theoretical models on the bases of the encoding-pattern hypothesis and processing-efficiency hypothesis (see Introduction). We performed a series of analyses on ERP and oscillatory power, with Position, Session, and Area as within-subject variables, separately for the MT and NC treatments. We expected the main effect of Session and/or interaction effect of Session and Position in the MT group.

#### ERP results

**Fig. 4:**
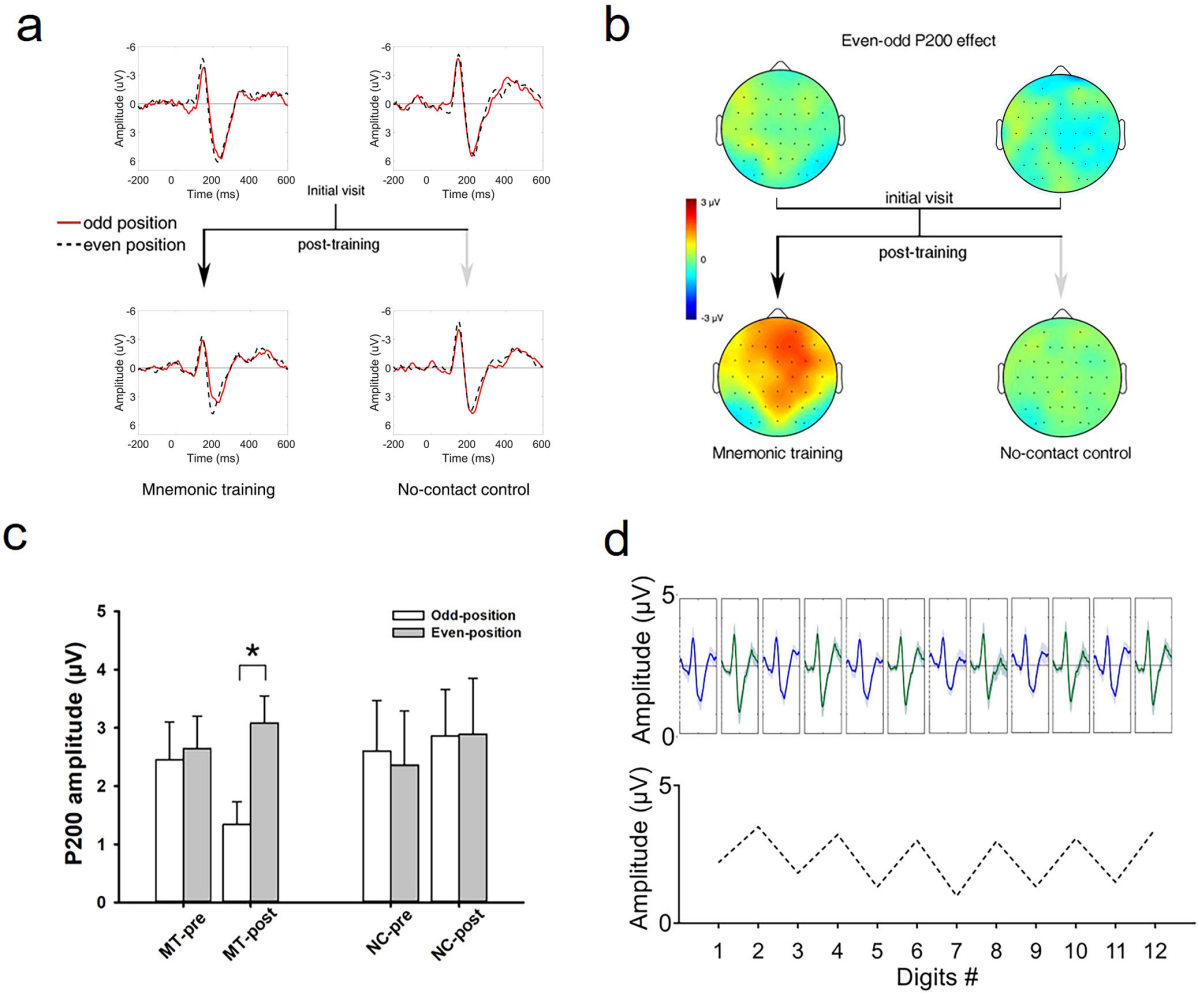
ERP results. **a** ERP waveforms (P200) averaged across all electrodes in the mnemonic training group (MT, *n* = 16) and the no-contact control group (NC, *n* = 17) are depicted separately at pre- and post-training. **b** Topographies of the P200 effect (even- vs. odd- positions) in two groups, depicted separately at pre- and post-training. **c** After training, the MT group demonstrates a significant increase in P200 amplitude from the odd- to the even-position condition. **d** *Upper*: example P200 odd-even pattern (0–400 ms windows) at FCz from a randomly selected 12-digit sequence in the MT-post. *Lower*: mean P200 amplitudes as a function of digit number. NC-pre/NC-post = noncontact group at pre-/post-training, MT-pre/MT-post = mnemonic training group at pre-/post-training. *p < 0.05, two-sided, paired-sample *t*-test. Error bars represent standard errors.

In the MT group, the main effect of Session failed to reach significance, *F* (1, 15) = 2.29, *p* = 0.15, *η*^*2*^_*partial*_ = 0.11. However, we detected a main effect of Position, *F* (1, 15) = 8.74, *p* < 0.01, *η*^*2*^_*partial*_ = 0.33, and its interaction with Area, *F* (8, 120) = 3.79, *p* < 0.05, *η*^*2*^_*partial*_ = 0.17. More importantly, we found the interaction effect of Position × Session, *F* (1, 15) = 3.79, *p* < 0.05, *η*^*2*^_*partial*_ = 0.17: the post-training session showed greater P200 amplitude in response to even-position digits (3.08 ± 0.96 μV) compared to odd-position digits (1.34 ± 0.80 μV), *p* < 0.05 (Fig. 4). No other main effect or interaction was significant. As a control, in the NC group, there was no such P200 effect, as indicated by the lack of significance for the effects involving Session or Position, all *p*s > 0.41.

#### Oscillatory power results

**Fig. 5:**
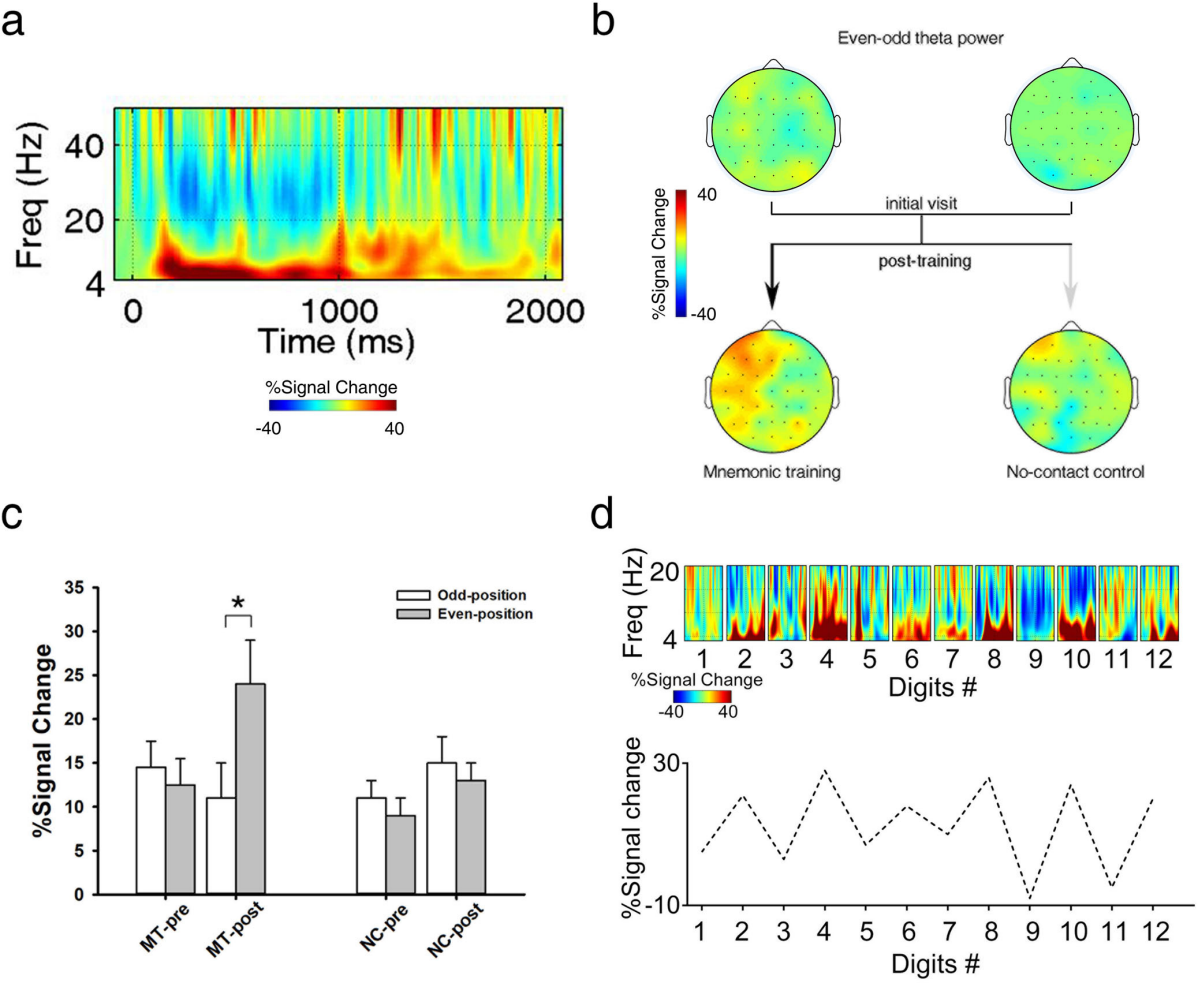
Oscillatory power results. **a** Frontal theta power (4–8 Hz) averaged across all frontal electrodes in the MT group (*n* = 16) at the post-training session. Red indicates more power for the even- versus odd-position condition. **b** Topographies of theta power (even- vs. odd- position, 200–1000 ms) in two groups. **c** At post-training, the MT group demonstrated a significant increase in theta power from the odd- to the even-position condition in the left anterior area. **d** *Upper*: example odd-even pattern of theta oscillations (0–1000 ms windows) at F3 from a randomly selected 12-digit sequence in the MT-post. *Lower*: mean theta power as a function of digit number. NC-pre/NC-post = noncontact group at pre-/post-training, MT-pre/MT-post = mnemonic training group at pre-/post-training. *p < 0.05, two-sided, paired-sample *t*-test. Error bars represent standard errors.

In the MT group, the main effect of Session was not significant, *F* (1, 15) = 2.16, *p* = 0.14, *η*^*2*^_*partial*_ = 0.15. However, the main effect of Position, *F* (1, 15) = 5.40, *p* < 0.05, *η*^*2*^_*partial*_ = 0.31, and the interaction effect of Area × Position, *F* (2, 30) = 3.82, *p* < 0.05, *η*^*2*^_*partial*_ = 0.24, reached significance. Importantly, there was a significant three-way interaction, *F* (2, 30) = 6.92, *p* < 0.05, *η*^*2*^_*partial*_ = 0.37. Breaking down the ANOVA based on the factor Session revealed an interaction between Position and Area for the post-training session, *F* (2, 30) = 14.43, *p* < 0.01, *η*^*2*^_*partial*_ = 0.55. Further analysis showed that the post-training session demonstrated a significant increase in theta power from the odd- (10 ± 5 %, percent signal change) to even-position (24 ± 6 %) condition in the left anterior area, *p* < 0.05 (Fig. 5), but the pre-training session did not. No other main effect or interaction was significant. In the NC group, the three-way ANOVA found no significant results, all *p*s > 0.09.

### Neural-behavioral relationships

**Fig. 6:**
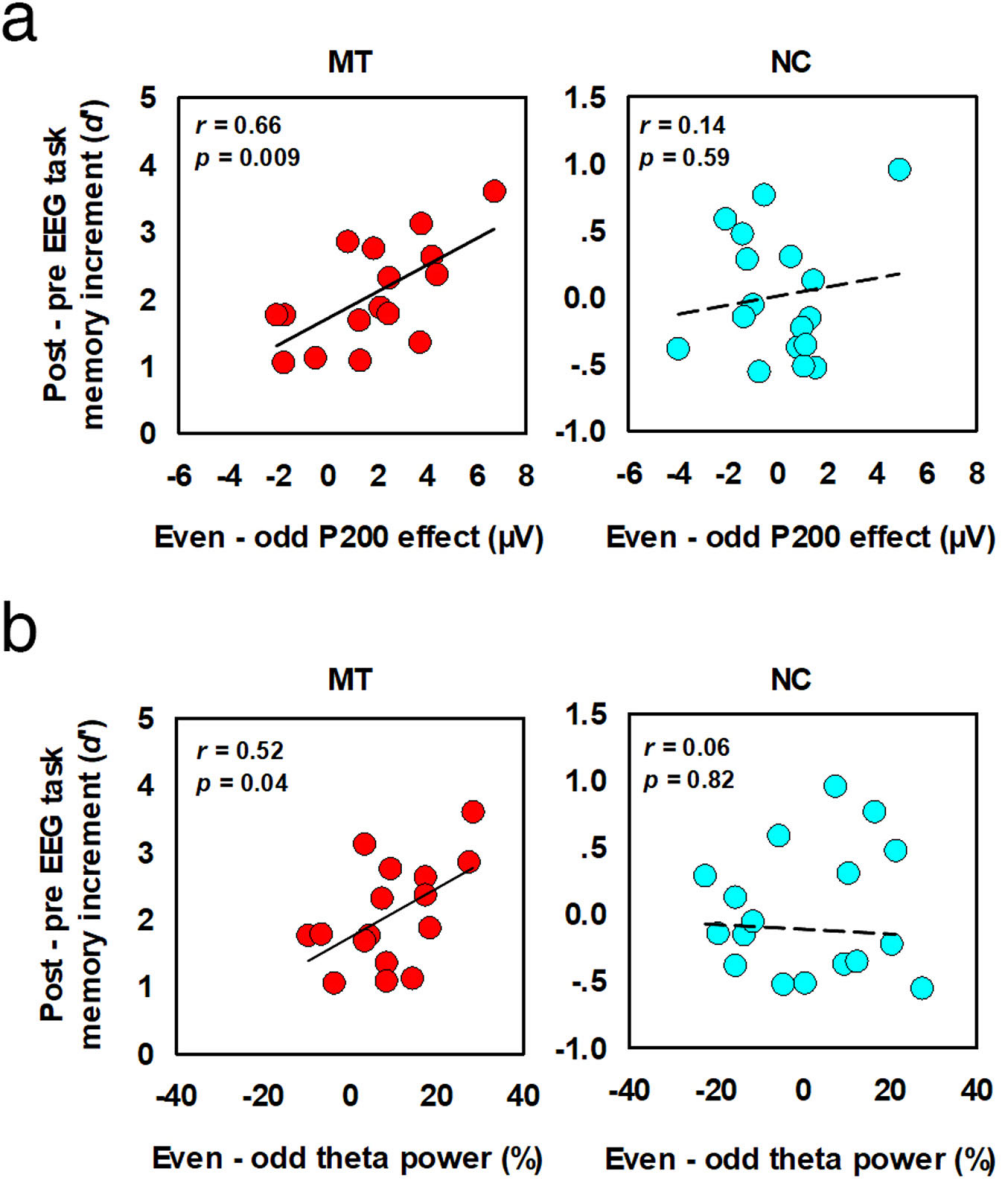
Neural-behavioral correlation. Scatter plots of EEG task memory increment (*d’*) and P200 effect (**a**) and theta power effect (**b**) in the MT and NC groups. Pearson correlation analyses were performed to estimate neural-behavioral relationships. MT, mnemonic training; NC, no-contact control.

To investigate the relationship between mnemonic training and enhanced memory, we calculated the Pearson correlations between neural activity and memory increment in the EEG task. Memory increment was indexed by the difference between *d’* at post-training and that at pre-training (*d’*_post_ - *d’*_pre_). The P200 (and theta power) effects were calculated by subtracting amplitudes (and power values) at odd-positions from those at even-positions (post-training evaluation). We found a significant correlation between the P200 effect (averaged over all significant electrodes) and memory increment in the MT group only (*r* = 0.66, *p* = 0.009, NC group: *r* = 0.13, *p* = 0.63; Fig. 6a). A Fisher’s *z* test^29^ showed that the correlation in MT was significantly larger than that in NC, *z* = 1.72, *p* = 0.04. Moreover, the left anterior frontal (F3 electrode) power effect significantly correlated with the memory increment in the MT group (*r* = 0.52, *p* = 0.04) but not in the NC group (*r* = 0.06, *p* = 0.82; Fig. 6b), z = 1.34, *p* = 0.09.

### Validation analyses

To further test whether there exist generally decreased/efficient neural activity after mnemonic training, two validation analyses were conducted in the MT group: (1) ERP validation. We went through significant ERP differences between pre- and post-training sessions across the entire time window (0–2000 ms, 0 ms indicates the stimuli onset) using a cluster-based method. Clusters were defined by window-by-window *t*-tests with a criterion of *p* < 0.05 (FDR corrected) for at least 10 consecutive time points and 4 adjacent electrodes; (2) oscillatory power validation. Cluster-based analysis was conducted to identify significant power differences between pre- and post-training sessions across the entire time window. This validation procedure was performed at three typical frequency bands: theta (4–8 Hz), alpha (9–12 Hz), and beta (13–30 Hz) bands. With ERP validation, no significant results survived after the cluster-based correction. However, with oscillatory power validation, we found a significant power difference between pre- and post-training sessions in the window of 1000–1400 ms at the alpha band (9–12 Hz). Further analysis detected that the post-training session elicited a more increased alpha power (18 ± 2 %) than the pre-training session (6 ± 3 %), *p*_*corr*_ < 0.05. No significant difference was found at other bands or in other time windows. In addition, the increased alpha power failed to significantly correlate with memory increment, *r* = 0.23, *p* = 0.57.

## Discussion

This study explored neural mechanisms underlying the mnemonic training effect on digit memory in developing children. Particularly, we tested whether this effect could be attributed to processing-efficiency and/or encoding-pattern. Our results provide valuable evidence supporting the latter account (in accordance with Model B, see Fig. 1). Specifically, after mnemonic training, the children demonstrated elevated P200 and increased theta power during encoding of even- vs. odd-position digits. Furthermore, such periodic neural patterns predicted a training-induced boost in digit memory.

Coupled with previous work, P200 was relevant to early attentional processing^30^, which was important for the formation of memory^31^. In the current study, the P200 effect was likely to be associated with the early attentional process of the use of mnemonic. Specifically, the MT group might recruit more top-down allocation of attentional resources to convert digits at the even-positions into pre-stored images, leading to larger P200^13^. In contrast, the NC group only had to attend to equally each digit itself, leading to no attentional bias. Apart from increased attention, several other cognitive processes may have contributed to the odd-/even-digit difference. One such process could be chunking^32^, which may have led to a stronger association between even-digits and the final image, as even-digits tend to form more meaningful units or chunks compared to odd digits. The mapping of digits to images may have also played a role^13^, as even digits may have been more easily mapped to concrete images due to their symmetry and balance. Furthermore, language-based retrieval might have influenced the encoding of even-digits^33^, as they could be verbalized and retrieved from memory. Mnemonic training also triggered changes in theta oscillatory power. Theta power during encoding has been shown to predict later memory^26,34^. Specifically, increases in theta band activity were associated with efficient encoding of the recognized vs. new items^35^ and the remembered vs. forgotten items^26^. The revealed frontal theta in the present study might also embody computational mechanisms for manipulating and monitoring items in working memory^36^. Moreover, we noted that theta activity was left-lateralized. Considering that verbal encoding appears to be strongly left-lateralized in the human brain^37^, this may indicate that MT participants relied on some verbal/semantic memory (e.g., story strategy) during encoding.

There are two other noteworthy points to consider. First, mnemonic training altered encoding pattern, as reflected in functional brain organization, at a very early stage (starting as early as 170 ms post stimuli, as reflected in P200). This finding echoed with previous studies that expertise-related processing of information can be initiated at a very early stage^38^. Second, it appears that a sustained period of processing, lasting approximately 800 ms as indicated by theta power, is required for the alteration of the encoding pattern. Such sustainability was reported to be associated with information preservation^13^ and items maintenance^39^. To sum up, mnemonic training in children may re-structure both early and sustained neural pattern, leading to better memory enhancement.

Our study provides evidence supporting the encoding-pattern hypothesis. We demonstrated the alteration of encoding pattern (as reflected in regular odd-even neural patterns), and the correlation between this organized pattern and memory increment. The functional alteration process after mnemonic training, reflected by the brain activation in left prefrontal^40^, inferior frontal cortex^41^, and functional connectivity within the visual, medial temporal lobe and default mode networks^4^, is important for the superior memory. Furthermore, supporting the organizational bias for training, research has shown the chunking and hierarchical neural activity in superior mnemonists^2,13^. Our findings were in accordance with previous studies showing the organizational processing of memory encoding after mnemonic training; for example, the posterior P200 differentiated primacy items from plateau portions^42^, and the frontal positive slow wave (PSW) distinguished successive semantically related words from non-related items^43^. A possible explanation of these variations is that organization of neural pattern in a list depends on how the mnemonic was used^44^. In Rushby et al.^42^, an elaborative mnemonic was used by semantically grouping three or five items; in Nogueira et al.^43^, a chunking mnemonic was used through clustering the three words in the middle serial positions; in the present study, a digit-image mnemonic was used by converting digit pairs into images on every even-position. Future studies should specifically examine the role of neural activity in supporting different types of the mnemonic.

One might argue that the odd-even patterns of EEG responses could simply reflect the constraints of the task: MT participants were taught to view the items in pairs and, in that sense, the procedure itself imposes the pattern on the trainee. Importantly, this issue does not apply to our study due to the following two reasons. First, MT participants were not explicitly asked to encode the digits in an odd-even manner. Both groups were instructed to memorize the materials as best as they could, without other constraints. Second, we observed that the regular odd-even brain activity (P200 and theta power effects) was positively co-varied with the memory increment, strongly indicating the functional contribution of the EEG responses. Taken as such, these results exclude the possibility that mnemonic-trained brain tuning to a regular odd-even pattern emerged as a consequence of the alignment between neural processes and task constraints.

Although we did not provide clear support for the processing-efficiency hypothesis, our current analyses were insufficient to exclude completely the possibility that processing-efficiency might have engaged in the encoding after mnemonic training. It is possible that the processing-efficiency are characterized by the efficient neural network in the brain, which may not be detected in the ERP and oscillatory power analyses. Future studies using the network analyses (e.g., brain phase synchrony^45^ or small-world networks^46^) may provide more evidence to address this issue. It is also possible that there are generally decreased/efficient brain activity after mnemonic training, however, it fails to survive thresholding in our study. Future independent replications, as well as further work using other mnemonics or paradigms, are needed.

A line of studies has demonstrated that mnemonic training can increase memory performance in both healthy people^47^ and patients^48^. For example, individuals using keyword mnemonics recalled more concrete words both immediately after learning and after a one-day time interval^47^. Mnemonic training effectively increased memory for object location associations in patients with mild cognitive impairment^48^. Nevertheless, benefits of mnemonic training were largely specific to tasks for which participants had been trained, with a very limited range of generalization^49^. Moreover, expertise is mostly domain-specific^50^. Consistent with these findings, our study demonstrated that the MT group compared with the NC group showed limited enhancement of cognitive abilities after training (see Table 1). It might be the case that the trainees failed in spontaneously using their newly learned mnemonic method in untrained tasks^51^.

This study has several limitations. First, the power of our statistical tests may have been limited by sample size, though we believe that our primary findings provide important insights into the cognitive processes underlying the digit-image mnemonic. We did not use any statistical methods to predetermine the sample size for our experiment; instead, we based it on preliminary assessments of superior mnemonists^13^. We recommend that future replications of this study aim to strengthen the current results by enlisting a larger number of participants. Second, participants were assigned to groups based on their interests, which may have resulted in differences in their skill profiles from the outset, such as their ability to learn digits. Despite attempting to match participants' ages, other background factors (e.g., their willingness or availability for the training camp) may have acted as confounding variables. While this would less likely affect our pre-post results, it could limit the generalizability of the findings to a broader population. Third, we observed a notable interaction effect between Position and Session in the MT (but not NC) group; although the lack of a Group effect is not conclusive evidence, we would like to highlight this observation. Indeed, we used a planned-contrast approach on EEG statistical analyses, performing ANOVAs with Position, Session, and Area as within-subject variables, separately for the MT and NC treatments. To clarify, the primary aim of this study was to test our theoretical models on the bases of encoding-pattern hypothesis and processing-efficiency hypothesis at the neural level in individuals who use the digit-image mnemonic rather than contrasting group differences (see Introduction). It is recommended that future replications improve statistical power to enable the examination of three- or four-way interactions and solidify the current findings.

Our study provides insights into the cognitive mechanisms underlying the processing of digit-image mnemonics, which are commonly used in education, training, and memory improvement (at least in some schools and institutes). Specifically, our results suggest that the encoding of even-odd digit pairs is effective in creating vivid and distinctive mental images, which can enhance memory performance. These findings have implications for educational and training programs that use digit-image mnemonics as a memory aid. Additionally, our study contributes to a better understanding of the role of attention and mental imagery in memory encoding, which has implications for the development of interventions and strategies to improve memory performance. Though our study did not detect clear transfer effect on a range of cognitive abilities, we believe that our findings can inform future research on the transfer effects of mnemonic strategies to other cognitive domains, such as problem-solving, decision-making, and creativity. For example, future studies may examine whether the use of digit-image mnemonics can enhance performance in these domains, and whether such effects are mediated by attention and mental imagery processes.

Taken together, this study tested the hypotheses that mnemonic training improves processing-efficiency and/or alters encoding-pattern to support memory enhancement. We provided evidence supporting encoding-pattern hypothesis by showing that mnemonic trainees exhibited regular odd-even neural patterns during processing even- versus odd- position digits after mnemonic training. Crucially, such odd-even neural pattern predicted better memory. This study thus constitutes direct electrophysiological evidence of how mnemonics alter encoding pattern, as reflected in functional brain organization, to support memory enhancement.

## Methods

### Participants

Forty-one children with no history of neurological disorders were recruited from primary or middle schools in Hainan, China (mean age: 12.97 years; 22 boys). None of them had previous experience with memory training. Twenty children were assigned to the mnemonic training group (MT group; age: 13.00 ± 2.19 years) and twenty-one to the no-contact control group (i.e., no contact during the 22-day training period; NC group; age: 12.94 ± 1.85 years) based on their interest in mnemonic training. There was no statistical difference between the two groups in terms of age, *t*(31) = 0.08, *p* = 0.93. Individuals who had too much body movement, as indicated by above 70% trials containing EEG amplitudes exceeding ± 100 μV (3 MT participants, 2 NC participants), or unable to insist in the tasks, as indicated by memory performance below 25% (1 MT participant, 1 NC participant), or met with apparatus problems (1 NC participant), were excluded. As a result, the data of 33 participants (MT: *n* = 16; NC: *n* = 17) were used in the behavior and EEG analyses. Each participant received CNY 100 for their participation. Our experimental procedures were conducted according to the Declaration of Helsinki and the research protocol was approved by the University Committee on Human Research Protection, East China Normal University. All participants and their parents provided written informed consent to take part in the study.

### Experimental procedure

This study had a longitudinal design with four sessions: (1) pre-training tests (initial visit), in which participants’ brain responses to digit sequences and their cognitive abilities were measured through an EEG task and a cognitive test battery, respectively; (2) training period (22 days), in which the MT group was trained to use the digit-image method on various materials; (3) post-training tests (immediately after training), in which training effects were assessed through the same tasks used in the initial visit; (4) follow-up tests (4 months after training), in which training effects on some measures (i.e., digit matrix and number-noun pairs) in the cognitive test battery were estimated again (Fig. 2a).

#### Pre-training tests (initial visit)

Both MT and NC groups participated in the tests. In the EEG task, participants studied and recalled 20 digit sequences, each as a single block including the following three phases (Fig. 2b): (*i*) *encoding phase*. Each digit initiated with a fixation cross (0.5 s), then a 2-s digit was presented, followed by an inter-stimulus interval (ISI, 0–0.5 s). 12 digits were presented one-by-one; (*ii*) *retention phase*. After the presentation of the 12-digit sequence, a blank screen for retention was shown (6 s); (*iii*) *retrieval phase*. A fixation (0.5 s) then alerted the beginning of retrieval, followed by a probe of the 2-digit number (1 s) and a screen of two letters (Y N), which remained until participants responded, or until 3 s had elapsed. Participants had to decide whether the probe was in the sequence just studied before, through a keyboard [“F” key for Y (yes), “J” key for N (no)]. For example, for a given sequence “241365984582” presented one-by-one during the encoding phase, if the probe displays “24”, participants should press “F”; if it shows “29”, they should press “J”. To note, only 2-digit combinations on the odd-even positions in the sequence were chosen, e.g., 24, 13; those with an even-odd order, such as 41, were not considered. For each sequence, there were 6 probes (half “yes” and half “no”). The discriminability [i.e., *d’*; *d’* = *z* (hit) – *z* (false alarm)] for all probes was used to evaluate their performance in the EEG task.

The cognitive test battery included tasks spanning the following abilities: perceptual speed, assessed by the two-choice-reaction tasks (digit and figure versions^6^); inhibitory control, assessed by the Stroop color task^52^; working memory, assessed by the 2-back tasks (digital and spatial 2-back versions^53^); short-term memory, assessed by the digit matrix memory task^11^; episodic memory, assessed by two tasks of studying word lists and number-noun pairs^6^; reasoning ability, assessed by the grammatical reasoning test^54^; spatial imagination, assessed by the Purdue visualization of rotations test^55^; and divided attention, assessed by the multi-object tracking task^56^.

#### Training period (three weeks)

The MT group participated in the World Memory Championships Training Camp, an institution aiming at training children to be superior mnemonists. The intense training lasted for 22 days with a minimum of 6 h per day. MT participants learned a conversion table of 100 unique images that were associated with 2-digit combinations from “00” to “99”. For example, “72” (/qī èr/) was encoded to 企鹅 (/qǐ é/, *penguin*) due to their phonological similarity in Chinese; “51” was encoded to 工人 (/gōng rén/, *worker*) due to the semantic association between labour (May 1st International Labour Day) and worker (Fig. 2a). They applied the digit-image method to practice memorizing digits (e.g., phone numbers) and materials used in the World Memory Championship competition, entailing Spoken Numbers, 30 min Binary Digits, 60 min Hour Numbers, and 5 min Random Numbers (for more details, see http://www.world-memory-statistics.com/).

#### Post-training tests (immediately after training)

The EEG task and cognitive test battery were tested again in both groups, with the same procedures but different materials.

#### Follow-up tests (4 months after training)

The digit matrix task and the number-noun pairs task from the cognitive test battery were chosen and tested again in 4 months to further assess the persistence of training effects.

### EEG data acquisition

EEG recordings were carried out using a 64-channel cap with the Neuroscan Synamps2 system (Compumedics), in accordance with the international 10/10 system. Vertical and horizontal electrooculograms (EOGs) were monitored and recorded as well: two electrodes were placed over and below the dominant eye for the vertical eye movements, and two electrodes were placed at the outer canthi of the eyes for the horizontal movements. The electrode impedance was kept under 10 kΩ. EEG data were digitized at 500 Hz, with an online bandpass filter from 0.05 to 100 Hz applied. An average mastoid reference was used.

### Behavioral data analysis

SPSS 16.0 (Chicago, IL, USA) was used to perform the statistical analyses. Repeated measures analyses of variance (ANOVAs), with the between-subject factor of Group (MT vs. NC) and a within-subject factor of Session (pre- vs. post-training), were conducted on participants’ discriminability (*d*’) in the EEG task and performance (accuracy or reaction time) in the cognitive test battery.

### EEG preprocessing

EEG data were pre-processed using EEGLAB (version 10.2.2.4^57^) and custom MATLAB (MathWorks Inc., Natick, MA) scripts. Signals were downsampled to 250 Hz and segmented (from −500 to 2000 ms relative to the stimulus onset). Eye-movement artifacts were removed using an independent component analysis (ICA) method. EEG data were then re-referenced to a common average reference. For the event-related potentials (ERPs) and oscillatory power analyses, the mean voltage from 500 ms preceding the onset of the digit was served as the baseline and subtracted from EEG data. Any noisy epochs with deflections greater than ± 100 μV were removed. For ERPs, EEG signals were low-passed filtered at 40 Hz. Trials were sorted according to the Position (even- vs. odd-positions, 20 digit-sequence * 6 even- or odd-positions = 120 trials in total). For the ERP and oscillatory power analyses, there were on average 90.4 trials (range: 71–118) and 84.2 trials (range: 62–108) per condition.

### ERP analysis

In line with previous work^13^, we focused on the P200. The selection of this component was confirmed by visual inspection of the waveforms and our preliminary analyses (i.e., examining ERPs for each participant and running *t*-tests to determine potential differences or similarities between groups). The mean of P200 was determined by the average value of the amplitude from a time window of 170–220 ms after the onset of each digit stimulus. For statistical analysis, all EEG electrodes were divided into different areas: left anterior (F3, F5, F7, FT7, FC3, FC5), medial anterior (F1, Fz, F2, FC1, FCz, FC2), right anterior (F4, F6, F8, FT8, FC4, FC6), left central (C3, CP3, C5, CP5, T7, TP7), medial central (C1, CP1, CPz, Cz, C2, CP2), right central (C4, CP4, C6, CP6, T8, TP8), left posterior (P7, P5, P3, PO7, PO3, O1), medial posterior (Pz, POz, Oz, P2), right posterior (P4, P6, P8, PO4, PO8, O2). The mean amplitudes of P200 were analyzed using repeated-measures ANOVAs. Specifically, to examine whether mnemonic training (MT) alters encoding-pattern or improves processing-efficiency or both at the neural level, we performed a series of planned ANOVA contrasts (focusing on training effects from the MT treatment). These comparisons were pre-specified based on our hypotheses – mnemonic training would improve processing-efficiency and/or alters encoding-pattern to support memory enhancement in developing children. A planned contrast also allowed us to test the training effect with a compromised sample size. Repeated-measures ANOVAs were conducted (separately for the MT and NC treatments), with the within-subject factors of Position (even- vs. odd- position), Session (pre- vs. post-training), and Area (left anterior vs. medial anterior vs. right anterior vs. left central vs. medial central vs. right central vs. left posterior vs. medial posterior vs. right posterior). The Bonferroni correction was applied for multiple comparisons in the planned ANOVA contrasts. Specifically, the alpha level was adjusted by dividing it by the number of post-hoc pairwise comparisons.

### Oscillatory power analysis

Event-related spectral perturbations (ERSP) were calculated using custom MATLAB code. We conducted a complex Morlet wavelet (five-cycle; lowest and highest frequency: 4 Hz and 30 Hz) to analyze oscillatory power. Frequencies from 4 to 30 Hz were divided by 27 linear frequency steps. Power values here represented a percentage increase/decrease of power relative to a −500 to 0 ms pre-stimulus baseline (i.e., digit onset). For each trial, we used time-frequency decomposition and then calculated the average power across all trials. Alike many previous studies that used the event-related spectral perturbations (ERSP) approach^58–60^, the evoked response power was not removed from the oscillatory response; however, given the time window and spatial distribution of the P200 and theta rhythm in our study, it is less likely that the results solely represent the phase-locked power. According to previous studies^26,35,61^, we focused on the frontal theta power (4–8 Hz, the mean power in the time window of 200–1000 ms). For statistical analysis, areas of frontal theta power were composited from the electrodes as follows: left anterior (F3, F5, F7, FT7, FC3, FC5), medial anterior (F1, Fz, F2, FC1, FCz, FC2) and right anterior (F4, F6, F8, FT8, FC4, FC6). Planned three-way repeated-measures ANOVAs were conducted (separately for the MT and NC treatments, see above), with factors of Position (even- vs. odd- position), Session (pre- vs. post-training), and Area (left anterior vs. medial anterior vs. right anterior) for the frontal theta oscillation. To account for multiple comparisons in the planned ANOVA contrasts, the Bonferroni correction was employed. The alpha level was adjusted by dividing it by the number of post-hoc pairwise comparisons. In line with previous work^13^, epochs were computed individually for each digit presented during the encoding phase, for both ERP and oscillatory power analyses.

### Reporting summary

Further information on research design is available in the Nature Research Reporting Summary linked to this article.

### Supplementary information

Supplement

Reporting Summary

## Data Availability

Restrictions apply to the availability of these data, which involve information about developing children and so are not publicly available. However, de-identified data that support the findings of this study are available from the corresponding author upon reasonable request for research purposes.

## Code availability

The data were analyzed using EEGLAB toolbox in MATLAB that is publicly accessible. Custom codes are available from the corresponding author upon reasonable request for research purposes.

## References

[1] Nielsen T (2013). The method of loci (MoL) and memory consolidation: Dreaming is not MoL-like. Behav. Brain Sci..

[2] Yin L-J, Lou Y-T, Fan M-X, Wang Z-X, Hu Y (2015). Neural evidence for the use of digit-image mnemonic in a superior memorist: an fMRI study. Front. Hum. Neurosci..

[3] Littlefield LM, Klein ER, Coates S (2012). An experimental evaluation of the effects of using training sentences to aide young children’s word recall. Eff. Educ..

[4] Dresler M (2017). Mnemonic Training Reshapes Brain Networks to Support Superior Memory. Neuron.

[5] Brehmer Y, Li S-C, Müller V, von Oertzen T, Lindenberger U (2007). Memory plasticity across the life span: Uncovering children’s latent potential. Dev. Psychol..

[6] Schmiedek F, Lövdén M, Lindenberger U (2010). Hundred days of cognitive training enhance broad cognitive abilities in adulthood: findings from the COGITO study. Front. Aging Neurosci..

[7] Neubauer AC, Fink A (2009). Intelligence and neural efficiency. Neurosci. Biobehav. Rev..

[8] Babiloni C (2010). “Neural efficiency” of experts’ brain during judgment of actions: A high-resolution EEG study in elite and amateur karate athletes. Behav. Brain Res..

[9] Herzmann G, Curran T (2011). Experts’ memory: an ERP study of perceptual expertise effects on encoding and recognition. Mem. Cogn..

[10] Andreasen NC (1995). PET Studies of Memory: Novel versus Practiced Free Recall of Word Lists. Neuroimage.

[11] Hu Y, Ericsson KA, Yang D, Lu C (2009). Superior self-paced memorization of digits in spite of a normal digit span: The structure of a memorist’s skill. J. Exp. Psychol. Learn. Mem. Cogn..

[12] Hu Y, Ericsson KA (2012). Memorization and recall of very long lists accounted for within the Long-Term Working Memory framework. Cogn. Psychol..

[13] Pan Y (2017). ERPs and oscillations during encoding predict retrieval of digit memory in superior mnemonists. Brain Cogn..

[14] Hanslmayr S (2008). The Electrophysiological Dynamics of Interference during the Stroop Task. J. Cogn. Neurosci..

[15] Shen X (2005). Sex Differences in perceptual processing: Performance on the color-kanji Stroop task of visual stimuli. Int. J. Neurosci..

[16] Ila AB, Polich J (1999). P300 and response time from a manual Stroop task. Clin. Neurophysiol..

[17] Jolles DD, Crone EA (2012). Training the developing brain: a neurocognitive perspective. Front. Hum. Neurosci..

[18] Swanson HL, Kehler P, Jerman O (2010). Working Memory, Strategy Knowledge, and Strategy Instruction in Children With Reading Disabilities. J. Learn. Disabil..

[19] Turley-Ames K (2003). Strategy training and working memory task performance. J. Mem. Lang..

[20] Astle DE, Barnes JJ, Baker K, Colclough GL, Woolrich MW (2015). Cognitive Training Enhances Intrinsic Brain Connectivity in Childhood. J. Neurosci..

[21] Everts, R., Mürner-Lavanchy, I., Schroth, G. & Steinlin, M. Neural change following different memory training approaches in very preterm born children – A pilot study. *Dev. Neurorehabil*. 1–11 10.3109/17518423.2015.1027010 (2015). 10.3109/17518423.2015.1027010 25905646

[22] Jonkman LM, Hurks PP, Schleepen TMJ (2016). Effects of memory strategy training on performance and event-related brain potentials of children with ADHD in an episodic memory task. Neuropsychol. Rehabil..

[23] Bottiroli S, Cavallini E, Dunlosky J, Vecchi T, Hertzog C (2013). The importance of training strategy adaptation: A learner-oriented approach for improving older adults’ memory and transfer. J. Exp. Psychol. Appl..

[24] Wu J, Mai X, Chan CCH, Zheng Y, Luo Y (2006). Event-related potentials during mental imagery of animal sounds. Psychophysiology.

[25] Herweg NA, Solomon EA, Kahana MJ (2020). Theta Oscillations in Human Memory. Trends Cogn. Sci..

[26] White TP (2013). Theta power during encoding predicts subsequent-memory performance and default mode network deactivation. Hum. Brain Mapp..

[27] de Borst AW (2012). Integration of “what” and “where” in frontal cortex during visual imagery of scenes. Neuroimage.

[28] Van der Lubbe RHJ, Sobierajewicz J, Jongsma MLA, Verwey WB, Przekoracka-Krawczyk A (2021). Frontal brain areas are more involved during motor imagery than during motor execution/preparation of a response sequence. Int. J. Psychophysiol..

[29] Diedenhofen B, Musch J (2015). cocor: A Comprehensive Solution for the Statistical Comparison of Correlations. PLoS One.

[30] Peters J, Suchan B, Zhang Y, Daum I (2005). Visuo-verbal interactions in working memory: Evidence from event-related potentials. Cogn. Brain Res..

[31] Gazzaley A, Nobre AC (2012). Top-down modulation: bridging selective attention and working memory. Trends Cogn. Sci..

[32] Thalmann M, Souza AS, Oberauer K (2019). How does chunking help working memory?. J. Exp. Psychol. Learn. Mem. Cogn..

[33] Craik FIM, Watkins MJ (1973). The role of rehearsal in short-term memory. J. Verbal Learn. Verbal Behav..

[34] Sederberg PB (2006). Oscillatory correlates of the primacy effect in episodic memory. Neuroimage.

[35] Osipova D (2006). Theta and Gamma Oscillations Predict Encoding and Retrieval of Declarative Memory. J. Neurosci..

[36] Petrides M, Tomaiuolo F, Yeterian EH, Pandya DN (2012). The prefrontal cortex: Comparative architectonic organization in the human and the macaque monkey brains. Cortex.

[37] Jansen A (2009). Assessment of verbal memory by fMRI: Lateralization and functional neuroanatomy. Clin. Neurol. Neurosurg..

[38] Wright MJ, Gobet F, Chassy P, Ramchandani PN (2013). ERP to chess stimuli reveal expert-novice differences in the amplitudes of N2 and P3 components. Psychophysiology.

[39] Curtis CE, D’Esposito M (2003). Persistent activity in the prefrontal cortex during working memory. Trends Cogn. Sci..

[40] Savage CR (2001). Prefrontal regions supporting spontaneous and directed application of verbal learning strategies: Evidence from PET. Brain.

[41] Bonner-Jackson A, Haut K, Csernansky JG, Barch DM (2005). The Influence of Encoding Strategy on Episodic Memory and Cortical Activity in Schizophrenia. Biol. Psychiatry.

[42] Rushby JA, Barry RJ, Johnstone SJ (2002). Event-related potential correlates of serial-position effects during an elaborative memory test. Int. J. Psychophysiol..

[43] Nogueira AML, Bueno OFA, Manzano GM, Kohn AF, Pompéia S (2015). Late positive slow waves as markers of chunking during encoding. Front. Psychol..

[44] Sanfratello L (2014). Same task, different strategies: How brain networks can be influenced by memory strategy. Hum. Brain Mapp..

[45] Lachaux J, Rodriguez E, Martinerie J, Varela FJ (1999). Measuring phase synchrony in brain signals. Hum. Brain Mapp..

[46] Smit DJA, Stam CJ, Posthuma D, Boomsma DI, de Geus EJC (2008). Heritability of “small-world” networks in the brain: A graph theoretical analysis of resting-state EEG functional connectivity. Hum. Brain Mapp..

[47] Campos A, Camino E, Pérez-Fabello MJ (2011). Using the Keyword Mnemonics Method Among Adult Learners. Educ. Gerontol..

[48] Hampstead BM (2012). Mnemonic strategy training improves memory for object location associations in both healthy elderly and patients with amnestic mild cognitive impairment: A randomized, single-blind study. Neuropsychology.

[49] Lustig C, Flegal KE (2008). Targeting latent function: Encouraging effective encoding for successful memory training and transfer. Psychol. Aging.

[50] Chase, W. G. & Ericsson, K. A. Skill and working memory. in *The psychology of learning and motivation* (ed. G. H. Bower) (Academic Press, 1982).

[51] McDaniel MA, Bugg JM (2012). Memory training interventions: What has been forgotten?. J. Appl. Res. Mem. Cogn..

[52] Golden, C. *Stroop Color and Word Test: A manual for clinical and experimental uses*. (Chicago: Stoelting, 1978).

[53] Cohen JD (1997). Temporal dynamics of brain activation during a working memory task. Nature.

[54] Baddeley AD (1968). A 3 min reasoning test based on grammatical transformation. Psychon. Sci..

[55] BODNER GM, GUAY RB (1997). The Purdue Visualization of Rotations Test. Chem. Educ..

[56] Spencer JP, Barich K, Goldberg J, Perone S (2012). Behavioral dynamics and neural grounding of a dynamic field theory of multi-object tracking. J. Integr. Neurosci..

[57] Delorme A, Makeig S (2004). EEGLAB: an open source toolbox for analysis of single-trial EEG dynamics including independent component analysis. J. Neurosci. Methods.

[58] Vecchio F (2022). Time-frequency analysis of brain activity in response to directional and non-directional visual stimuli: an event related spectral perturbations (ERSP) study. J. Neural Eng..

[59] Gu H, Chen Q, Xing X, Zhao J, Li X (2019). Facial emotion recognition in deaf children: Evidence from event-related potentials and event-related spectral perturbation analysis. Neurosci. Lett..

[60] Possti D (2021). Changes in the EEG spectral power during dual-task walking with aging and Parkinson’s disease: initial findings using Event-Related Spectral Perturbation analysis. J. Neurol..

[61] Onton J, Delorme A, Makeig S (2005). Frontal midline EEG dynamics during working memory. Neuroimage.

