# Supplementary material for: Mnemonic-trained brain tuning to a regular odd-even pattern subserves digit memory in children
Source: NPJ Sci Learn. 2023 Aug 11;8:27. doi: 10.1038/s41539-023-00177-8 (PMC10421878; doi:10.1038/s41539-023-00177-8)
Supplement: Supplementary file 1 — Supplement [file 41539_2023_177_MOESM1_ESM.docx]

**Supplementary Results**

For the performance on our cognitive test battery, we conducted a series of ANOVAs, with Group as a between-subject factor and Session as a within-subject factor. The results of main and interaction effects were summarized in **Supplementary** **Table 1**. Since many measures were used, we applied false-discovery-rate (FDR) multiple-comparison corrections. Importantly, we observed a significant interaction effect on the number-noun pairs measure, suggesting that the MT group showed better performance on this test compared to the NC group after training and this showed long-term effects in the follow-up.

**Supplementary Table 1.** The results of ANOVAs for each measure of cognitive test battery.

| Test battery | Group | | Session | | Group × Session  (four months later) | |
| --- | --- | --- | --- | --- | --- | --- |
|  | *F* | *p* | *F* | *P* | *F* | *p* |
| Perceptual speed (ms) |  |  |  |  |  |  |
| *Choice reaction-digit* | 0.15 | 0.70 | 0.99 | 0.33 | 1.64 | 0.21 |
| *Choice reaction-figure* | 0.09 | 0.76 | 44.35 | **<0.001** | 0.15 | 0.70 |
| Inhibitory control (ms) |  |  |  |  |  |  |
| *Stroop color* | 0.00 | 0.98 | 6.55 | 0.02 | 0.05 | 0.83 |
| Working memory (accuracy) |  |  |  |  |  |  |
| *Digital 2-back* | 3.83 | 0.06 | 4.82 | 0.04 | 0.18 | 0.67 |
| *Spatial 2-back* | 1.30 | 0.26 | 3.40 | 0.08 | 0.00 | 0.99 |
| Short-term memory (accuracy) |  |  |  |  |  |  |
| *Digit matrix* | 12.73 | **0.002** | 5.59 | **0.007** | 0.60 | 0.55 |
| Episodic memory (accuracy) |  |  |  |  |  |  |
| *Word lists* | 17.35 | **<0.001** | 2.03 | 0.16 | 3.42 | 0.07 |
| *Number-noun pairs* | 16.00 | **0.001** | 4.55 | 0.02 | 5.22 | **0.009** |
| Reasoning ability (accuracy) |  |  |  |  |  |  |
| *Reasoning* | 2.54 | 0.12 | 3.74 | 0.06 | 0.45 | 0.51 |
| Spatial imagination (accuracy) |  |  |  |  |  |  |
| *Rotations* | 0.03 | 0.86 | 0.00 | 0.98 | 0.10 | 0.75 |
| Divided attention (accuracy) |  |  |  |  |  |  |
| *Multi-object tracking* | 0.13 | 0.72 | 8.53 | **0.007** | 0.06 | 0.82 |

*Note*. Bolded *p* values survived after FDR corrections (*p*_FDR_ < 0.05).
