# Supplementary material for: Mnemonic-trained brain tuning to a regular odd-even pattern subserves digit memory in children
Source: NPJ Sci Learn. 2023 Aug 11;8:27. doi: 10.1038/s41539-023-00177-8 (PMC10421878; doi:10.1038/s41539-023-00177-8)
Supplement: Supplementary file 2 — Reporting Summary [file 41539_2023_177_MOESM2_ESM.pdf]

nature portfolio

Corresponding author(s): Yixuan Ku, Yi Hu

Last updated by author(s): May 11, 2023

Reporting Summary

Nature Portfolio wishes to improve the reproducibility of the work that we publish. This form provides structure for consistency and transparency in reporting. For further information on Nature Portfolio policies, see our Editorial Policies and the Editorial Policy Checklist.

Statistics

For all statistical analyses, confirm that the following items are present in the figure legend, table legend, main text, or Methods section.

n/a	Confirmed	
☐	☒	The exact sample size (*n*) for each experimental group/condition, given as a discrete number and unit of measurement
☐	☒	A statement on whether measurements were taken from distinct samples or whether the same sample was measured repeatedly
☐	☒	The statistical test(s) used AND whether they are one- or two-sided<br>*Only common tests should be described solely by name; describe more complex techniques in the Methods section.*
☒	☐	A description of all covariates tested
☐	☒	A description of any assumptions or corrections, such as tests of normality and adjustment for multiple comparisons
☐	☒	A full description of the statistical parameters including central tendency (e.g. means) or other basic estimates (e.g. regression coefficient) AND variation (e.g. standard deviation) or associated estimates of uncertainty (e.g. confidence intervals)
☐	☒	For null hypothesis testing, the test statistic (e.g. *F*, *t*, *r*) with confidence intervals, effect sizes, degrees of freedom and *P* value noted<br>*Give P values as exact values whenever suitable.*
☒	☐	For Bayesian analysis, information on the choice of priors and Markov chain Monte Carlo settings
☒	☐	For hierarchical and complex designs, identification of the appropriate level for tests and full reporting of outcomes
☐	☒	Estimates of effect sizes (e.g. Cohen's *d*, Pearson's *r*), indicating how they were calculated

Our web collection on statistics for biologists contains articles on many of the points above.

Software and code

Policy information about availability of computer code

Data collection	EEG recordings were carried out using a 64-channel cap with the Neuroscan Synamps2 system (Compumedics), in accordance with the international 10/10 system.
Data analysis	SPSS 16.0 (Chicago, IL, USA) was used to perform the behavioral statistical analyses. The EEG data were analyzed using EEGLAB toolbox in MATLAB that is publicly accessible.

For manuscripts utilizing custom algorithms or software that are central to the research but not yet described in published literature, software must be made available to editors and reviewers. We strongly encourage code deposition in a community repository (e.g. GitHub). See the Nature Portfolio guidelines for submitting code & software for further information.

Data

Policy information about availability of data

All manuscripts must include a data availability statement. This statement should provide the following information, where applicable:

- Accession codes, unique identifiers, or web links for publicly available datasets
- A description of any restrictions on data availability
- For clinical datasets or third party data, please ensure that the statement adheres to our policy

De-identified data available from the corresponding author, upon reasonable request.

Research involving human participants, their data, or biological material

Policy information about studies with human participants or human data. See also policy information about sex, gender (identity/presentation), and sexual orientation and race, ethnicity and racism.

Reporting on sex and gender	Forty-one children were recruited (22 boys and 19 girls).
Reporting on race, ethnicity, or other socially relevant groupings	All participants were Chinese.
Population characteristics	Twenty children were assigned to the mnemonic training group (MT group; age: 13.00 ± 2.19 years) and twenty-one to the no-contact control group (i.e., no contact during the 22-day training period; NC group; age: 12.94 ± 1.85 years) based on their interest in mnemonic training. There was no statistical difference between the two groups in terms of age, $t(31) = 0.08$, $p = 0.93$.
Recruitment	Forty-one children with no history of neurological disorders were recruited from primary or middle schools in Hainan, China
Ethics oversight	The research protocol was approved by the University Committee on Human Research Protection, East China Normal University.

Note that full information on the approval of the study protocol must also be provided in the manuscript.

Field-specific reporting

Please select the one below that is the best fit for your research. If you are not sure, read the appropriate sections before making your selection.

☒ Life sciences ☐ Behavioural & social sciences ☐ Ecological, evolutionary & environmental sciences

For a reference copy of the document with all sections, see nature.com/documents/nr-reporting-summary-flat.pdf

Life sciences study design

All studies must disclose on these points even when the disclosure is negative.

Sample size	We did not use any statistical methods to predetermine the sample size for our experiment; instead, we based it on preliminary assessments of superior mnemonists (Pan et al., 2017).
Data exclusions	Individuals who had too much body movement, as indicated by above 70% trials containing EEG amplitudes exceeding ± 100 µV (3 MT participants, 2 NC participants), or unable to insist in the tasks, as indicated by memory performance below 25% (1 MT participant, 1 NC participant), or met with apparatus problems (1 NC participant), were excluded.
Replication	No replication has been conducted yet. But our current findings on the mnemonic training group partly replicated our previous findings on superior mnemonists (Pan et al., 2017).
Randomization	Participants were assigned to groups based on their interests, which may have resulted in differences in their skill profiles from the outset, such as their ability to learn digits. Despite attempting to match participants' ages, other background factors (e.g., their willingness or availability for the training camp) may have acted as confounding variables. While this would less likely affect our pre-post results, it could limit the generalizability of the findings to a broader population. We have explicitly referred to this point as a limitation in our manuscript.
Blinding	Blinding was not possible as the mnemonic training group participated in a summer camp while the no-contact control group did not.

Reporting for specific materials, systems and methods

We require information from authors about some types of materials, experimental systems and methods used in many studies. Here, indicate whether each material, system or method listed is relevant to your study. If you are not sure if a list item applies to your research, read the appropriate section before selecting a response.

Materials & experimental systems

n/a	Involved in the study
☒	☐ Antibodies
☒	☐ Eukaryotic cell lines
☒	☐ Palaeontology and archaeology
☒	☐ Animals and other organisms
☒	☐ Clinical data
☒	☐ Dual use research of concern
☒	☐ Plants

Methods

n/a	Involved in the study
☒	☐ ChIP-seq
☒	☐ Flow cytometry
☒	☐ MRI-based neuroimaging
